# Development and validation of a risk prediction model for overall survival in cervical cancer patients under 50: a prospective cohort study in southwest China

**DOI:** 10.7717/peerj.20509

**Published:** 2026-01-05

**Authors:** Li Yuan, Baogang Wen, Xiuying Li, Fangrong Liu, Haike Lei, Qi Zhou

**Affiliations:** 1 Chongqing University Cancer Hospital, ChongQing University, Chongqing, China; 2 Chongqing Cancer Multi-omics Big Data Application Engineering Research Center, Chongqing University Cancer Hospital, Chongqing, China

**Keywords:** Cervical cancer, Prediction model, Nomogram, Prognosis, Overall survival

## Abstract

**Objective:**

Accurately predicting the prognosis of cervical cancer in younger patients is increasingly important due to the rising incidence of the disease in China and the growing number of cases among individuals under 50. This study aimed to develop a nomogram to predict overall survival (OS) in cervical cancer patients under 50 in southwest China.

**Methods:**

Clinicopathological and follow-up data for cervical cancer patients under 50 were prospectively collected as part of an ongoing longitudinal cohort study at Chongqing University Cancer Hospital between January 1, 2015, and May 31, 2019. A training cohort (*n* = 703) and a validation cohort (*n* = 301) were randomly selected. Variables associated with OS were assessed using a Cox regression model. Multivariate analysis was used to construct the nomogram and identify independent prognostic factors. The model’s performance was evaluated using decision curve analysis (DCA), calibration curves, area under the receiver operating characteristic curve (AUC-ROC), and the concordance index.

**Results:**

The final model identified pathology, International Federation of Gynecology and Obstetrics (FIGO) staging, treatment, β2-microglobulin, neutrophil-lymphocyte ratio (NLR), and albumin as independent risk factors for OS in patients under 50. The concordance index for OS was 0.818 in the training cohort and 0.747 in the validation cohort. Calibration curves in both cohorts showed strong agreement between predicted and observed survival probabilities. In the training cohort, AUCs for 1-, 3-, and 5-year OS were 0.851, 0.847, and 0.816, respectively; in the validation cohort, they were 0.810, 0.733, and 0.730. Compared to the FIGO staging system, the nomogram demonstrated superior predictive accuracy and net benefit, as shown by the net reclassification index (NRI) and DCA.

**Conclusion:**

The nomogram provides a reliable tool for predicting overall survival in cervical cancer patients under 50, supporting more personalized treatment planning.

## Introduction

Cervical carcinoma is the predominant neoplasm of the female reproductive system and is the fourth commonest cancer in women, following lung, colorectal, and breast cancers. Annually, over 604,127 new cases and 341,831 mortalities have been reported around the world (Singh et al., 2023). Subsequently breast cancer, cervical carcinoma is the second most often diagnosed malignant tumor among Chinese women. Cervical cancer-related mortality appears to be slightly higher in rural areas than in urban areas in China (Yuan et al., 2023). Cervical cancer is most prevalent in two age groups: the most prevalent ranges from the ages of 40 and 50, and the subsequent peak is among the ages of 60 and 70. It is unusual to develop cervical cancer before the age of 20. The median incidence of cervical cancer is 51 years old. It is noteworthy, although, that there has been a gradual decline in the average age at which cervical cancer first appears in recent years, with a tendency toward young (Siegel, Giaquinto & Jemal, 2024). Human papillomavirus (HPV) vaccination has been reported to reduce the occurrence of pathologic changes in the cervix. Many women in low-resource nations have an invasive diagnosis of disease in the absence of efficient cervical cancer screening programs (Petersen et al., 2022). However, communities with inadequate services with a greater prevalence of cervical cancer and worse outcomes can exist in even highly resourced nations. The optimal treatment choice hinges on the International Federation of Gynecology and Obstetrics (FIGO) staging. Precisely defining the anatomical scope of illness and forecasting the chances of survival are the key components of an effective staging system. The staging of cervical cancer is perpetually refined due to technological developments that improve the diagnosis as well as treatment (Wright et al., 2019). The treatment option for early-stage cervical carcinoma is radical hysterectomy combined with pelvic lymphadenectomy. However, around 25% of patients who undergo this radical surgery experience recurrences. Concurrent chemoradiotherapy is the preferable treatment for patients with high-risk factors, including positive surgical margins, parametrial infiltration and positive lymph nodes. The selection of adjuvant treatment is guided by the Sedlis criteria among individuals with medium-risk features, such as lymph vascular space invasion, large tumor size and deep stromal invasion (Wolswinkel et al., 2023). Because the FIGO staging method is a clinical staging system that is not based on pathologic findings, it has the inherent disadvantage of being unable to make specific prognosis forecasts (Bhatla et al., 2021). In fact, in a lot of cases, the pathologic condition does not correspond to the FIGO stage. A clinical staging system that is not predicated on pathological findings is the FIGO staging method. Consequently, it has the inherent disadvantage of being unable to make specific prognosis predictions. Actually, in numerous cases, the pathologic state frequently does not correspond to the FIGO staging system. Thus, in order to complement the FIGO staging system, it is important to develop an additional predictive model.

Nomograms are widely used in oncology and other medical fields as predictive tools. Nomograms meet our need for clinically and biologically integrated models and advance personalized treatment by combining a variety of prognostic and determinant characteristics to fulfill our desire for personalized medical treatment (Zheng et al., 2017). The use of nomogram-derived prognosis for assisting clinical decision-making can be achieved with ease thanks to user-friendly digital interfaces that allow for quick computation, enhanced accuracy, and more understandable prognoses compared to traditional staging (Balachandran et al., 2015). In the field of oncology, nomograms have gained widespread recognition in recent years as effective predictive tools. It simplifies prognosis prediction for medical professionals, outlines the requirements for an integrated model, and contributes to the advancement of personalized medicine. The current prognostic model is suboptimal due to a multitude of factors, such as the radicality of the surgery, the number of patients included, the variable decision-making approach employed, and variations in adjuvant therapy regimens (Wang et al., 2018). All independent predictors of the course of a disease should be included in an ideal prediction model (Shipe et al., 2019). As such, developing a useful and reliable prognostic model is crucial for determining treatment options and assessing the survival probabilities of patients with cervical cancer. In order to support clinical decision-making, nominal staging enables the smooth integration of nomogram-derived prognosis. Within our analytical research, we developed nomogram models to provide quantitative probability and present a straightforward diagram of clinical events.

Predictive indicators have been developed with validation to forecast the probability of overall survival (OS) in a cohort of 1,004 patients under 50 years old diagnosed with cervical cancer at Chongqing University Cancer Hospital. The combination of these nomograms is expected to be helpful for medical decision-making and further research. These nomograms that combined are predicted to be beneficial for further research as well as medical decision-making. Crucially, we used the following methods to prognosticate OS and evaluate our model both internally and externally: calibration plots, decision curve analysis (DCA), C-index and receiver operating characteristic (ROC) curves. Our research is significantly more comprehensive and accurate than its predecessors due to these characteristics. The creation of a new, more precise prediction model is deemed essential for accurate prognosis evaluation and the improvement of treatment methodologies.

## Materials & Methods

### Origin of data

This prospective cohort research depends on the malignant tumor database platform of the cancer hospital at Chongqing University, including all patients under fifty years of age who were diagnosed with a new diagnosis of cervical carcinoma at the hospital since 2015. Patients who were admitted to the hospital between January 1, 2015 and May 31, 2019 are included in the patient cohort. We collected demographic information including gender, year of birth, ethnicity, period of diagnosis, and medical insurance, in combination with clinical data comprising pathological type and FIGO staging. The staging of cervical cancer was determined based on the International Federation of Gynecology and Obstetrics (FIGO) 2009 classification criteria. Treatment modalities including surgical intervention, radiation, chemotherapy, targeted treatment, and immunotherapy. Laboratory markers systematically documented included HPV infection status, lymphocyte count (LYM), neutrophil-lymphocyte ratio (NLR), platelet-lymphocyte ratio (PLR), neutrophil count, albumin/globulin ratio (A/G ratio), albumin concentration, β2-microglobulin levels and follow-up information.

**Figure 1:**
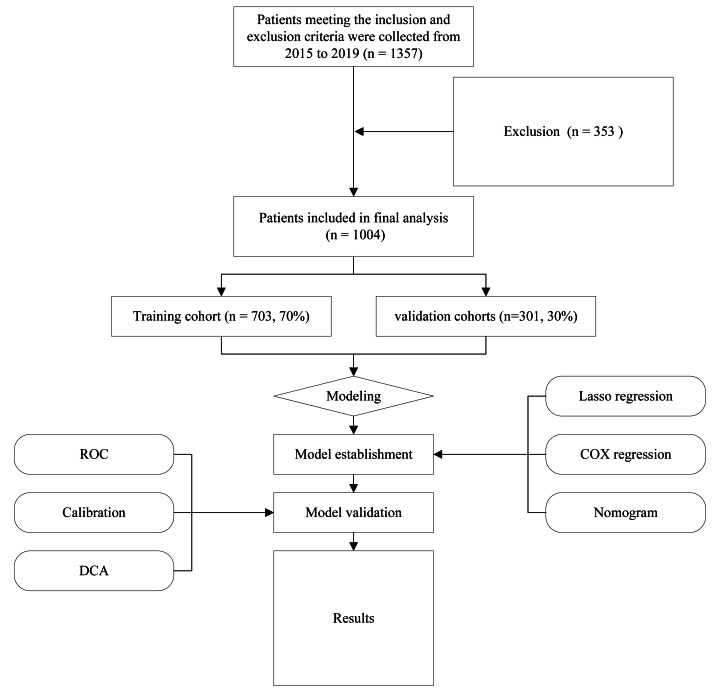
Flow diagram of study design.

The following criteria were considered for inclusion: (1) Patients over 50 who received their initial treatment at Chongqing University Cancer Hospital and was just diagnosed with cervical cancer; (2) comprehensive clinical information, including clinical diagnoses, pathological data, treatment strategies, and follow-up details; and (3) demographics of the patients and medical expense data. The following were the criteria for exclusion: (1) people with cervical cancer who are not newly diagnosed or those that did not undergo standard treatment; (2) cancer treatment history and follow-up data lacking. Figure 1 shows the flowchart for the research. The research received approval from the ethical committees of Chongqing University Cancer Hospital (CZLS2023015-A). Every participant willingly and knowingly provided their written consent.

### Variable

The demographic characteristics associated with the study population included were ages, marital relationship (married or unmarried), race (Han or other), and job position. Clinical staging has been established with regard to guidelines set by the FIGO staging system, using pathological data and metastatic data as clinical variables. The following information has been gathered: information regarding various treatment modalities, including surgical intervention, radiotherapy, chemotherapy, targeted therapy, immunotherapy, and blood test biomarkers.

### Developing a nomogram

Patients under 50 years of age were assigned at random to two distinct groups: a validation cohort (301 observations, about 30% of the dataset) and a training cohort (703 observations, nearly 70% of the dataset). The nomogram model was developed through the data from the training cohort. The study used a statistical method called univariate Cox regression analysis to identify the factors that could predict OS. Each covariate was analyzed and selected variables were those according to *p*-values of 0.05. These selected variables were then entered by the use of a multivariate analysis in order to determine independent risk factors for OS. The nomogram was created using risk ratings derived from the final Cox regression model, which was produced using a systematic stepwise approach.

### Validation and model performance

The accuracy of the nomogram analysis model was evaluated based on four methodologies: decision curve analysis (DCA), calibration curve, concordance index (C-index) as well as area under the receiver operating characteristic curve (AUC). The C-index is a measure of how accurately the model predicts outcomes, by comparing predicted and actual values. The effectiveness of a prediction model was assessed by comparing its predictions to the actual outcomes using a calibration curve. DCA followed in order to determine the medical “net benefit” associated with employing the model in comparison to standard treatment approaches for either all or none of the patients under 50 years of age.

### Follow-up and primary outcomes

Follow-up is the ongoing evaluation of patient prognostic data by phone or WeChat communication. The main outcomes of the research were the likelihood of OS at one, three, and five years.

### Development and verification of a clinical prediction model

The nomogram is the most commonly used technique for prognostic assessment in clinical research. It is a computational or comprehensive graphical computing approach that considers both biological and clinical data. The present research established and validated a nomogram to forecast OS in patients under 50 diagnosed with cervical cancer. The information collected were used to create and evaluate a medical model that predicts the survival probability of cervical carcinoma. Initially, variables within the training cohort were selected using the least absolute shrinkage and selection operator (LASSO) Cox regression model. Dummy variables were created for categorical factors. After conducting cross-validation, LASSO regression was used to identify the most important variables and confirm the appropriate tuning parameters (*λ*). This multivariate Cox proportional hazards analysis has been conducted using these characteristics. The Cox regression model results were used to develop a nomogram that predicts the probabilities of overall survival after one, three, and five years. This nomogram was assessed in a validation cohort for its predictive capability regarding survival outcomes, concentrating on discrimination and calibration assessments. The objective of constructing the calibration curve was to assess the model’s efficacy to differentiate between various outcomes and its predictive accuracy. The net reclassification index (NRI) and Integrated Discrimination Improvement (IDI) of the nomogram have been determined to evaluate its enhancement of the FIGO Staging system model with regard to of accuracy and predictive capability. Furthermore, DCA has been used to assess the potential therapeutic value of the prediction models.

### Statistical analysis

Descriptive statistics for continuous variables with a normal distribution are presented as means ± standard deviations, and intergroup differences were assessed using independent-samples t-tests. For continuous variables that were not normally distributed, data are presented as medians with interquartile ranges (IQRs), and group comparisons were performed using nonparametric tests. The Pearson Chi-square test was used to assess significant differences in demographic and clinical factors between the validation and training groups. Notable characteristics have been discerned by multivariate Cox regression analysis and LASSO regression. Through Cox Snell residual testing, it was found that the Cox regression in this study satisfies the PH assumptions. A web-based application has been developed with the Shiny and DynaNom packages are used to predict individual and dynamic rates of survival for patients through a nomogram (http://www.shinyapps.io/). Statistical analyses were performed using SPSS software version 26.0 (IBM Corporation, Armonk, NY, USA) and R software version 4.2.1 (R Core Team, 2022).

## Results

### Baseline characteristic

#### Characteristics of the training and validation cohorts

The clinical characteristics of the 1,004 patients under 50 years of age diagnosed with cervical cancer included in this research has been outlined in Table 1. Cervical cancer patients undergoing follow-up visits were assigned at random to training (*n* = 703, 70%) and validation (*n* = 301, 30%) groups utilizing the cancer database platform of Chongqing University Cancer Hospital. In generally, a significant percentage of the cohort belonged to the Han ethnicity (985, 98.11%), were married (933, 92.93%), had been diagnosed with squamous carcinoma (840, 83.67%), were at Stage I (592, 58.96%), and demonstrated HPV infection (598, 59.56%). There was a mean age of 43.0 ± 5.78 years among the patients.

**Table 1:** Demographics and clinical features of patients.

Variables	level	Overall	Training	validation	*p*
n		1,004	703	301	
Age (mean (SD))		43.024 (5.778)	42.983 (5.694)	43.120 (5.979)	0.7315
Marital (%)	Married	933 (92.93)	661 (94.03)	272 (90.37)	0.0526
	Others	71 (7.07)	42 (5.97)	29 (9.63)	
Ethnic (%)	Han	985 (98.11)	687 (97.72)	298 (99.00)	0.2669
	Minority	19 (1.89)	16 (2.28)	3 (1.00)	
Medicainsurance (%)	URBMI	721 (71.81)	511 (72.69)	210 (69.77)	0.3865
	UEBMI	283 (28.19)	192 (27.31)	91 (30.23)	
Pathological (%)	Squamous carcinoma	840 (83.67)	584 (83.07)	256 (85.05)	0.431
	Adenocarcinoma	126 (12.55)	94 (13.37)	32 (10.63)	
	Adeno acathoma/others	38 (3.78)	25 (3.56)	13 (4.32)	
Stage (%)	I	592 (58.96)	426 (60.60)	166 (55.15)	0.2248
	II	189 (18.82)	130 (18.49)	59 (19.60)	
	III–IV	223 (22.21)	147 (20.91)	76 (25.25)	
HPV (%)	Negative	406 (40.44)	291 (41.39)	115 (38.21)	0.3827
	Positive	598 (59.56)	412 (58.61)	186 (61.79)	
Radiotherapy (%)	No	639 (63.65)	459 (65.29)	180 (59.80)	0.1128
	Yes	365 (36.35)	244 (34.71)	121 (40.20)	
Chemotherapy (%)	No	627 (62.45)	453 (64.44)	174 (57.81)	0.0553
	Yes	377 (37.55)	250 (35.56)	127 (42.19)	
Targeted (%)	No	944 (94.02)	666 (94.74)	278 (92.36)	0.1898
	Yes	60 (5.98)	37 (5.26)	23 (7.64)	
Immunity (%)	No	997 (99.30)	697 (99.15)	300 (99.67)	0.6202
	Yes	7 (0.70)	6 (0.85)	1 (0.33)	
b2.microglobulin (mean (SD))		1.905 (0.988)	1.888 (1.079)	1.944 (0.736)	0.4192
A.G (mean (SD))		1.461 (0.287)	1.469 (0.296)	1.444 (0.264)	0.2147
NLR (mean (SD))		3.084 (2.243)	3.107 (2.158)	3.030 (2.433)	0.6145
PLR (mean (SD))		168.852 (92.929)	169.371 (91.199)	167.640 (96.993)	0.787
N1 (mean (SD))		4.381 (2.114)	4.367 (2.137)	4.415 (2.063)	0.7403
LYM (mean (SD))		1.594 (0.507)	1.577 (0.509)	1.632 (0.501)	0.1154
Alb (mean (SD))		43.648 (4.858)	43.681 (4.831)	43.569 (4.928)	0.7379

#### Training group independent predictive factors

Table 2 displays the results of the Cox proportional hazards models that were employed to identify independent predictive variables in the training cohort (*n* = 703). The following variables were identified as significant predictors of OS in the univariable analysis: Pathological, FIGO staging, Chemotherapy, β2-microglobulin, neutrophil-lymphocyte ratio (NLR) and albumin (Alb). The integration of pathology into the model was based on clinical consensus and previous research. In multivariable analysis, it has been found that pathology (adenosquamous carcinoma/others *vs.* squamous carcinoma and adenocarcinoma, HR: 7.56; 95% CI [3.49–16.37], *p* < 0.001), FIGO staging (III–IV *vs.* I and II, HR: 6.03; 95% CI [3.52–10.34], *p* < 0.001), chemotherapy (HR: 0.30; 95% CI [0.17–0.52], *p* < 0.001), β2-microglobulin (HR: 1.25; 95% CI [1.10–1.41], *p* < 0.001), NLR (HR: 1.17; 95% CI [1.10–1.24], *p* < 0.001), and Alb (HR: 0.91; 95% CI [0.87–0.96], *p* < 0.001) were significant independent predictors in multivariable analysis.

**Table 2:** Univariate and multivariate Cox regression analyses for OS in the training cohort.

Variables		all	HR (univariable)	HR (multivariable)	HR (final)
Age	Mean ± SD	43.0 ± 5.7	0.98 (0.95–1.02, *p* = .350)	0.98 (0.94–1.01, *p* = .196)	
Marital	Married	661 (94.0%)			
	Others	42 (6.0%)	1.10 (0.45–2.72, *p* = .834)	1.11 (0.41–2.99, *p* = .842)	
Ethnic	Han	687 (97.7%)			
	Minority	16 (2.3%)	1.15 (0.28–4.68, *p* = .845)	0.65 (0.14–3.11, *p* = .592)	
Medicainsurance	URBMI	511 (72.7%)			
	UEBMI	192 (27.3%)	0.85 (0.52–1.38, *p* = .504)	0.82 (0.49–1.40, *p* = .472)	
Pathological	Squamous carcinoma	584 (83.1%)			
	Adenocarcinoma	94 (13.4%)	0.67 (0.32–1.39, *p* = .286)	0.99 (0.46–2.14, *p* = .985)	0.93 (0.44–1.96, *p* = .844)
	Adeno acathoma/others	25 (3.6%)	3.21 (1.54–6.67, *p* = .002)	7.15 (3.20–15.96, *p* < .001)	7.56 (3.49–16.37, *p* < .001)
FIGO staging	I	426 (60.6%)			
	II	130 (18.5%)	2.54 (1.44–4.50, *p* = .001)	2.20 (1.20–4.01, *p* = .010)	2.11 (1.18–3.80, *p* = .012)
	III–IV	147 (20.9%)	6.37 (3.87–10.50, *p* < .001)	5.76 (3.25–10.20, *p* < .001)	6.03 (3.52–10.34, *p* < .001)
HPV	Negative	291 (41.4%)			
	Positive	412 (58.6%)	0.95 (0.62–1.46, *p* = .821)	0.85 (0.54–1.35, *p* = .497)	
Radiotherapy	No	459 (65.3%)			
	Yes	244 (34.7%)	0.74 (0.44–1.24, *p* = .250)	1,128,014.46 (0.00–Inf, *p* = .995)	
Chemotherapy	No	453 (64.4%)			
	Yes	250 (35.6%)	0.72 (0.43–1.21, *p* = .216)	0.00 (0.00–Inf, *p* = .995)	0.30 (0.17–0.52, *p* < .001)
β2.microglobulin	Mean ± SD	1.9 ± 1.1	1.55 (1.39–1.74, *p* < .001)	1.29 (1.12–1.48, *p* < .001)	1.25 (1.10–1.41, *p* < .001)
A/G ratio	Mean ± SD	1.5 ± 0.3	0.30 (0.14–0.64, *p* = .002)	1.63 (0.74–3.61, *p* = .224)	
NLR	Mean ± SD	3.1 ± 2.2	1.18 (1.13–1.25, *p* < .001)	1.05 (0.90–1.23, *p* = .498)	1.17 (1.10–1.24, *p* < .001)
PLR	Mean ± SD	169.4 ± 91.2	1.00 (1.00–1.01, *p* < .001)	1.00 (1.00–1.00, *p* = .158)	
N1	Mean ± SD	4.4 ± 2.1	1.08 (1.02–1.15, *p* = .007)	1.04 (0.92–1.17, *p* = .541)	
LYM	Mean ± SD	1.6 ± 0.5	0.44 (0.28–0.69, *p* < .001)	0.78 (0.43–1.43, *p* = .423)	
Alb	Mean ± SD	43.7 ± 4.8	0.88 (0.85–0.92, *p* < .001)	0.91 (0.86–0.95, *p* < .001)	0.91 (0.87–0.96, *p* < .001)

### Variable selection

**Figure 2:**
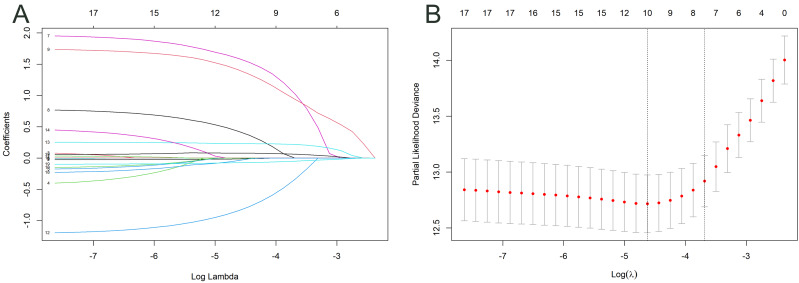
Log(lambda) sequence and LASSO model. A coefficient profile plot was created against the log(lambda) sequence (A). After confirming the optimal parameter (lambda) in the LASSO model, the partial likelihood deviance (binomial deviance) curve was plotted *versus* log(lambda), and dotted vertical lines were constructed based on one standard error criterion (B).

Two processes were used to select variables for the nomogram. For feature selection, the training cohort was initially exposed to the LASSO regression approach. The optimal tuning parameter for LASSO regression has been defined as 0.010, corresponding to the minimal partial probability binomial deviation (Fig. 2A). Consequently, the optimal lambda was utilized to choose factors with nonzero coefficients (Fig. 2B).

#### Prognostic nomogram model development

**Figure 3:**
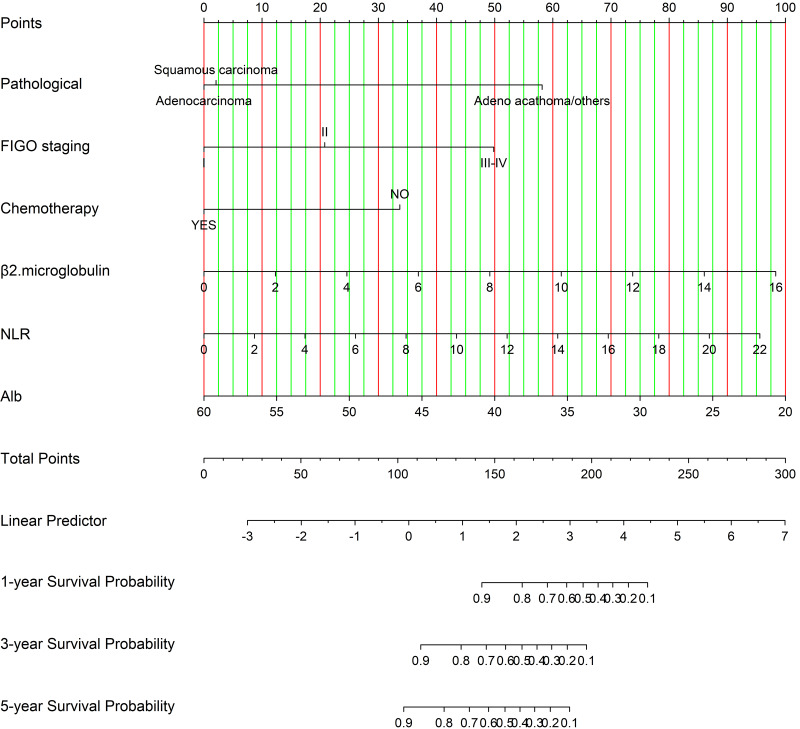
Nomogram for predicting one-year, three-year, and five-year overall survival in cervical cancer patients.

Figure 3 illustrates a nomogram and dynamic nomogram developed from the training cohort data for predicting survival probabilities in cervical cancer patients under 50 years of age. n order to create a nomogram model that can predict the OS of patients diagnosed with cervical cancer under the age of 50 for 1, 3, and 5 years, independent indicators that were demonstrated through multivariable analysis were chosen. The relevant Cox regression coefficients have been utilized for assigning a point score to each variable. The OS probability was subsequently calculated by accumulating the allocated points according to the total point table. Furthermore, we developed an online calculation tool for developing the nomogram model (https://cqcervical.shinyapps.io/cqcervical/) that improves its applicability. Users can automatically compute a patient’s survival probability by entering the relevant indicator values into the calculator.

#### Model performance and nomogram validation

**Figure 4:**
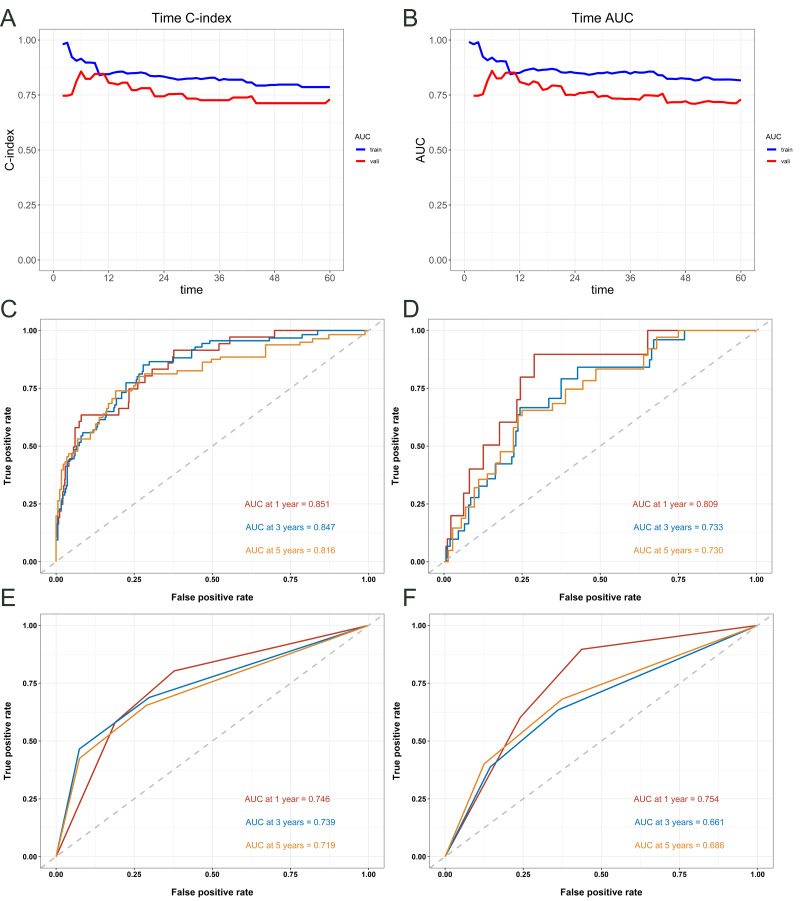
Evaluation of the predictive performance of the nomogram and FIGO staging system for overall survival in cervical cancer patients. Time- dependent concordance index (C- index) (A) and area under the curve (AUC) (B) for the nomogram model. ROC curves of the nomogram for predicting 1-, 3-, and 5- year OS in the training cohort (C) and validation cohort (D). ROC curves of the FIGO staging system for predicting 1-, 3-, and 5- year OS in the training cohort (E) and validation cohort (F).

Figure 4 provides an assessment of the predictive accuracy of the survival model for patients, measured by the C-index and area under the curve (AUC) at various time points (Figs. 4A–4B). The nomogram exhibited accordance indices (C-indices) of 0.816 (95% CI [0.774–0.858]) that predicted OS in cervical carcinoma sufferers under 50 years old within the training cohort, and the validation cohort’s 0.754 (95% CI [0.677–0.831]) showed remarkable discriminating ability. The receiver operating characteristic (ROC) values for predicting OS at 1-, 3-, and 5- years in the training cohort were 0.851, 0.847, and 0.816, respectively (Fig. 4C). The ROC values in the validation cohort were 0.809, 0.733, and 0.730 (Fig. 4D). In the training cohort, the ROC curves used to predict 1-, 3-, and 5-year overall survival according to FIGO Stage revealed values of 0.746, 0.739, and 0.719 (Fig. 4E), respectively. In the validation cohort, the curves yielded values of 0.754, 0.661, and 0.686 (Fig. 4F).

**Figure 5:**
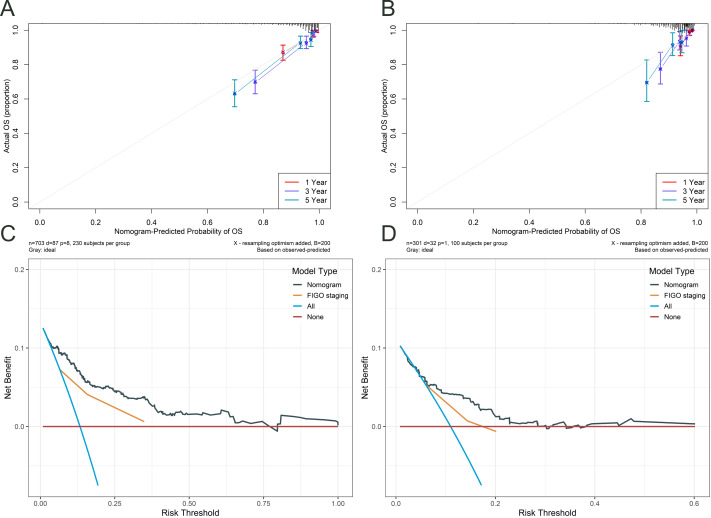
Calibration and DCA curves for the nomogram. The calibration curves for the training cohort (A) and testing cohort (B) demonstrated the agreement between the predicted and observed OS probabilities at 1-, 3-, and 5- year time points. Decision curve analysis (DCA) was performed to assess the clinical utility of the nomogram in predicting 5- year OS in cervical cancer patients, both in the training (C) and testing (D) cohorts.

Figure 5 illustrates the assessment of the nomogram’s predictive performance regarding patients aged under 50 with cervical cancer who have an OS, as well as an analysis of the net benefit of the nomogram-based predictions across various risk thresholds. Both the training cohort’s (Fig. 5A) and validation cohort’s (Fig. 5B) calibration curves for the 1-, 3-, and 5-year overall survival rates exhibit robust concordance between the predicted and observed probability. The figure also compares the nomogram’s predictions with those based solely on FIGO staging (Figs. 5C and 5D). The nomogram offers a comprehensive approach that is more nuanced and personalized in its assessment of OS probabilities than the FIGO staging system. The net benefit of the nomogram surpasses that of FIGO staging at all time points, indicating that the nomogram’s personalized predictions are more clinically beneficial.

**Figure 6:**
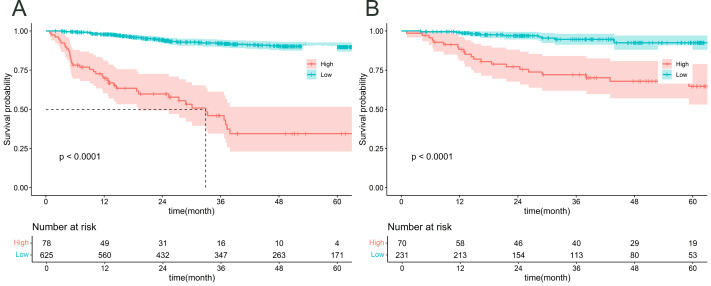
K–M curve analysis of risk stratification in the training (A) and validation (B) cohorts.

Figure 6 illustrates an analysis of OS in patients under the age of 50 who have been diagnosed with cervical cancer, with patients being classified into high-risk and low-risk categories according to the prediction model. Based on these predicted risk scores generated by our model, Patients were divided into high-risk and low-risk groups for both the training (Fig. 6A) and validation (Fig. 6B) cohorts. Subsequently, we constructed Kaplan–Meier survival curves to analyses the survival lengths between the two cohorts. The results indicated significant differences in survival times between the high-risk and low-risk groups in both cohorts (*p* < 0.05). These findings suggest that our model accurately predicts survival probabilities and effectively differentiates between high- and low-risk patients, thereby enhancing its potential clinical applications.

## Discussion

Cancer is usually thought of as an illness mostly impacting the elderly demographic, namely those aged 50 years and older. Emerging statistics indicate a significant increase in the occurrence of several organ malignancies among persons under 50, a condition termed early-onset cancer (Koh et al., 2023). These malignancies have a significant impact on a variety of organ systems, such as the breast, colon and/or rectum, pancreas, head and neck, kidney, and reproductive system. Mortality and morbidity are substantial consequences of early-onset cancer. In recent years, there has been a trend of cervical cancer occurring more frequently in younger individuals. Cervical cancer mortality demonstrated an increasing trend among women under 50 years of age in Kanagawa and across Japan from 1975 to 2012 (Motoki et al., 2015). Despite the existence of standardized treatment protocols for cervical cancer, patients frequently encounter the challenge of disease recurrence post-treatment (Burmeister et al., 2022). Upon recurrence, patients often face severely limited therapeutic alternatives and a bleak prognosis for the future (Chao et al., 2021). An accurate prediction is crucial for post-therapy follow-up and counselling for patients in cervical cancer. Precise prognostic prediction is crucial for providing personalized consultations, reporting health status, developing diagnoses, creating treatment strategies, and instituting follow-up procedures (Yang et al., 2020). Due to the limitations of the current FIGO staging system, the design of a novel prognostic prediction technology is essential. In response to the aforementioned challenges, we created a nomogram based upon real-world cases in order to forecast the survival rates of cervical cancer patients under 50 years of age, with the objective of providing personalized and precise prognoses. The data used to construct this nomogram were sourced from multiple clinical cases rather than a database, allowing for a more comprehensive inclusion of relevant predictors. Compared to multicenter and database-driven studies, data from single-center sources demonstrate high consistency and standardized treatment protocols. This standardization reduces the influence of human factors on study findings and emphasizes the importance of varied clinical evidence and pathological elements in prognosis by minimizing inconsistencies in treatment methods and subjective evaluation criteria among surgeons and pathologists across various institutions and nations. This study focuses on patients under the age of 50 diagnosed with cervical cancer, utilizing a prediction model based on a large sample from a single center and incorporating a wide range of clinical characteristics. The variables included in the nomogram, such as pathology type, surgical approach, radiotherapy, chemotherapy, HPV infection status, and hematological data, reflect greater innovation compared to previous prediction models. In our prospective cohort study, we assessed the clinical, pathological, therapeutic, and hematological attributes, as well as the prognosis, of 1,004 cervical cancer patients aged under 50. We developed a clinical prediction model to predict the OS rate for women under 50 diagnosed with cervical cancer using sixteen factors. The prognostic model incorporated clinically relevant factors, including age, marital status, ethnicity, medical insurance, pathological type, FIGO staging, HPV infection status, radiotherapy, chemotherapy, PLR, NLR, A/G ratio, N1, β2-microglobulin, LYM, and Alb. We identified pathologic type, FIGO staging, chemotherapy, Alb, NLR, and β2-microglobulin as independent prognostic factors through univariable and subsequent multivariable analysis.

The commonest histological subtypes are squamous cell carcinoma (SCC), adenocarcinoma (AC), and adenosquamous carcinoma (ASC) comprising a rarer variety that combines glandular and squamous components. Notably, the prevalence of cervical AC has risen dramatically in recent decades, particularly among younger individuals (Meng et al., 2021). However, the prognostic significance and treatment responses among cervical cancer patients under the age of 50 with varying histological subtypes remain inconclusive. Our study’s subgroup analysis found that among cervical cancer patients under the age of 50, squamous cell carcinoma had the best prognosis, whereas adenosquamous carcinoma had the worse.

FIGO staging is essential for evaluating the extent of tumor spread in gynecologic oncology, offering a comprehensive assessment of cancer stage (Bhatla et al., 2021). Consistent with this, our study demonstrated a negative correlation between FIGO staging and OS, reflecting the association between higher stages and increased disease progression.

In the current treatment of cervical cancer patients under the age of 50, various modalities such as surgical resection, chemotherapy, radiotherapy, and immunotherapy have shown promising outcomes in improving prognosis. Our study found that patients receiving concurrent chemoradiotherapy had significantly better prognoses than those who received radiotherapy alone without chemotherapy. This highlights the importance of personalizing treatment plans to optimize outcomes for individual patients.

β2-microglobulin, a light chain member of HLA-I, is produced by nearly all nucleated cells. It plays a crucial role in carcinogenesis and immunological control as a significant component of the major histocompatibility complex (MHC) class I (Wang, Liu & Wei, 2021). The multivariate analysis in our study identified β2-microglobulin as an independent prognostic determinant for cervical cancer patients under the age of 50, emphasizing its significant role in the nomogram model.

The neutrophil-to-lymphocyte ratio (NLR), commonly used to evaluate systemic inflammation and immune status, is increasingly recognized as a negative prognostic factor for clinical outcomes (Platini et al., 2022). The systemic balance between lymphocyte-mediated anti-tumor immune response and neutrophil-driven pro-tumor inflammation is assessed using the NLR (Sehgal et al., 2023). A shift toward greater pro-tumor inflammation and less anti-tumor immune function may be indicated by elevated NLR levels. NLR may have prognostic importance for patients with different forms of cancer, according to mounting data. The validity of NLR as a prognostic biomarker for cervical cancer patients under 50 has been further demonstrated by these retrospective investigations, which are consistent with the findings of our prospective research.

Albumin acts as an important medical marker reflecting nutritional status and liver synthesis capacity. Low albumin levels can impair immune function by affecting the metabolism and activity of immune cells. Previous studies have shown that albumin regulates the inflammatory response and plays an antioxidant role in tumor development. An elevated inflammatory response to tumor cells and an increased release of a variety of cancer-related cytokines that are implicated in tumor initiation are associated with a reduced serum albumin level (Oymak, Guler & Onal, 2023). Additionally, low serum albumin indicates malnutrition and serves as a marker of a weakened immune system in cancer sufferers. In this research, albumin was found as a significant predictor of treatment results for cervical cancer patients under the age of 50.

In our analysis, 1,004 patients under the age of 50 with invasive cervical cancer were included. Oncological outcomes were assessed using clinical, hematological parameters and pathological, all of which independently influence prognosis. Our study has notable limitations. First, the model was developed using data from a single hospital, which may limit its generalizability due to regional variations in the burden of cervical cancer patients under the age of 50. Second, the model has not yet undergone external validation, so additional prospective cohort studies using external data are needed to confirm the accuracy of our findings. Currently, we are actively involved in collaboration with multiple centers to obtain external datasets, intending to validate the model in more populations and clinical settings. Third, competing risks, such as deaths unrelated to cervical cancer, were not explicitly accounted for in the survival analysis. While this limitation may affect the accuracy of absolute risk estimates, the prognostic factors identified in our study remain valid for patient risk stratification. Future research should incorporate competing risk regression models to generate more precise cumulative incidence estimates and enhance the clinical applicability of the findings. Finally, while the study incorporated serum biomarkers, tumor type, and stage, it did not include other prognostic variables such as tumor size, lymph node status, lymphovascular space invasion (LVSI), depth of stromal invasion, or genomic data. Incorporating these factors in future studies could enhance the model’s comprehensiveness, predictive accuracy, and clinical applicability. Further research need to include these elements to improve the comprehensiveness and robustness of the research.

## Conclusions

We developed a prediction nomogram for the OS of cervical cancer patients under the age of 50, demonstrating excellent discrimination and calibration. This model provides a straightforward accurate method for forecasting the survival of patients, hence improving the capacity to customize therapies for the benefit of each patient.

##  Supplemental Information

10.7717/peerj.20509/supp-1 Supplemental Information 1 Original data

## Additional Information and Declarations

### Data Availability

The following information was supplied regarding data availability: The raw data is available in the Supplemental File.
